# Supplementary material for: Simplicial and topological descriptions of human brain dynamics
Source: Netw Neurosci. 2021 Jun 3;5(2):549–68. doi: 10.1162/netn_a_00190 (PMC8233107; doi:10.1162/netn_a_00190)
Supplement: Supplementary file 1 [file netn-05-549-s001.pdf]

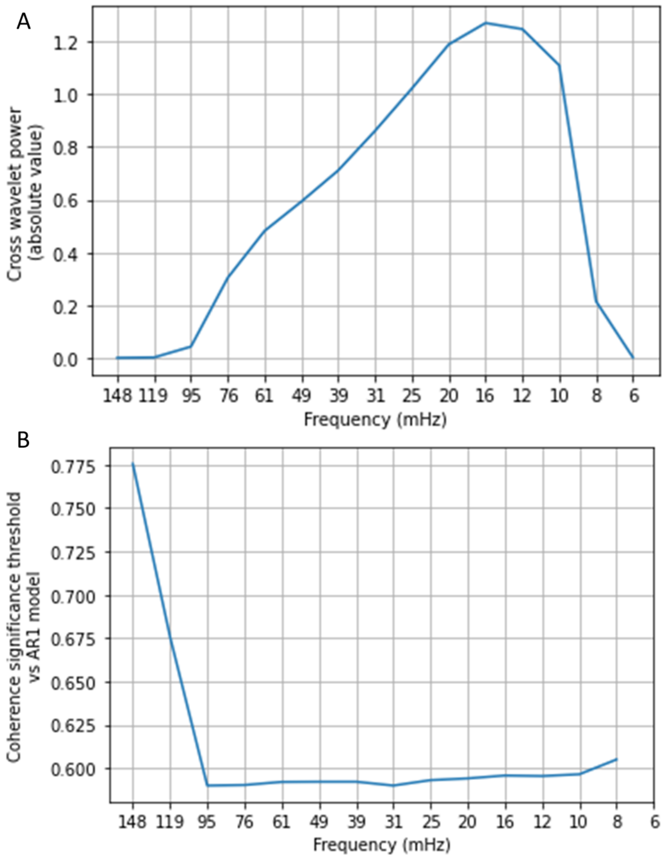

Figure 0.1: **Describes the mean values of the input data across frequency bands.** Part A of the figure displays the absolute value of the mean cross wavelet power. Frequencies between 0.01 and 0.02 Hz are most likely to contribute to the mean wavelet coherence used to quantify TVFC edges. Part B of the figure displays a significance threshold level indicating significantly high coherence. The threshold was calculated as an average against the background power spectrum. A distribution over the background power spectrum was calculated from 300 lag-1 approximations of each time series. Following Torrence and Compo (1998), the 95% confidence interval is the product of the background power spectrum and the 95th percentile value of a chi-squared distribution with two degrees of freedom.

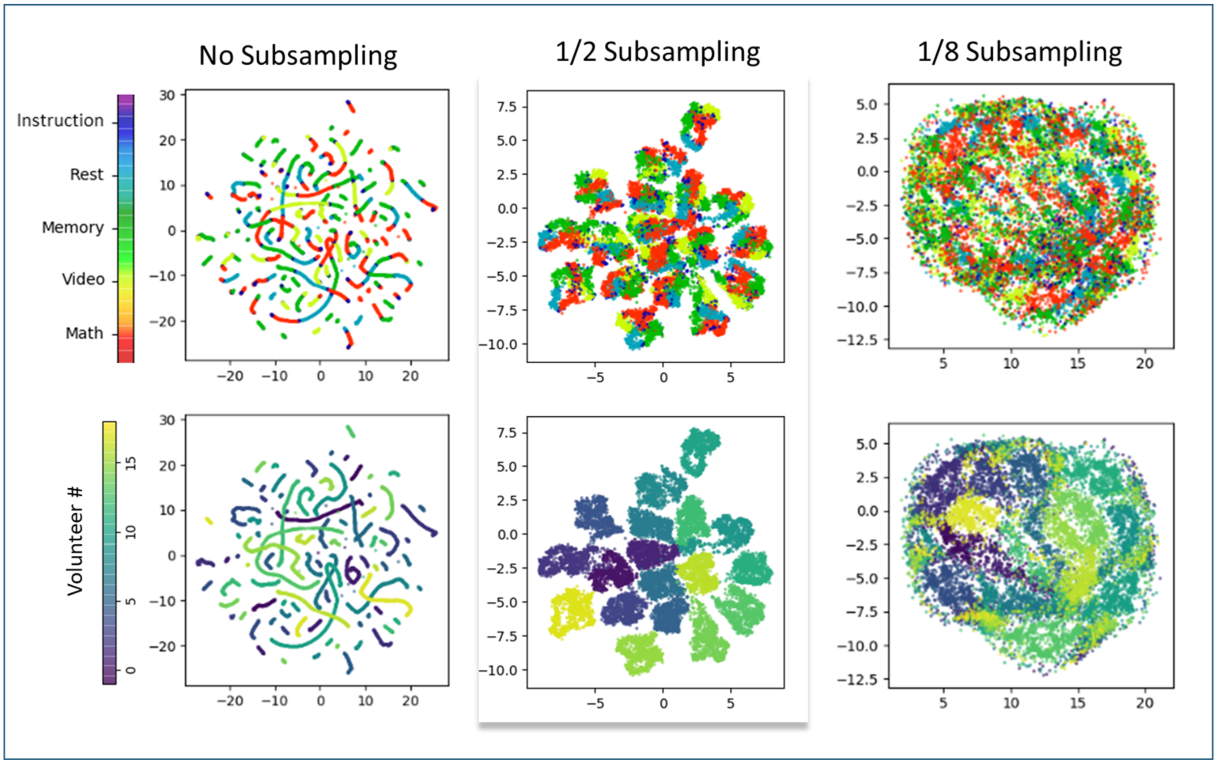

Figure 0.2: **Observes an embedding of the weighted Jaccard distance between edges at several levels of subsampling.** With no subsampling, successive time points from within the same scan retain strong attractive forces in the UMAP nearest-neighbor embedding. This produces string-like masses. Subsampling the data by half retains the similarities present within each volunteer's scan, thereby grouping volunteers into their own cluster. A noticeable degree of volunteerwise clustering—e.g., temporal self-similarity—is still present when seeding the embedding with 1/8 the total number of data points. None-the-less, some mutual attraction does emerge among edge-centric brain states measured across multiple volunteers.
